# Unsupervised abnormality detection in neonatal MRI brain scans using deep learning

**DOI:** 10.1038/s41598-023-38430-0

**Published:** 2023-07-17

**Authors:** Jad Dino Raad, Ratna Babu Chinnam, Suzan Arslanturk, Sidhartha Tan, Jeong-Won Jeong, Swati Mody

**Affiliations:** 1 Industrial and Systems Engineering Department, Wayne State University, Detroit, MI 48201 USA; 2 Computer Science Department, Wayne State University, Detroit, MI 48201 USA; 3 Department of Pediatrics, Wayne State University, Detroit, MI 48201 USA; 4 Division of Pediatric Radiology, University of Michigan, Ann Arbor, MI 48109 USA

**Keywords:** Computational science, Neurological disorders

## Abstract

Analysis of 3D medical imaging data has been a large topic of focus in the area of Machine Learning/Artificial Intelligence, though little work has been done in algorithmic (particularly unsupervised) analysis of neonatal brain MRI’s. A myriad of conditions can manifest at an early age, including neonatal encephalopathy (NE), which can result in lifelong physical consequences. As such, there is a dire need for better biomarkers of NE and other conditions. The objective of the study is to improve identification of anomalies and prognostication of neonatal MRI brain scans. We introduce a framework designed to support the analysis and assessment of neonatal MRI brain scans, the results of which can be used as an aid to neuroradiologists. We explored the efficacy of the framework through iterations of several deep convolutional Autoencoder (AE) unsupervised modeling architectures designed to learn normalcy of the neonatal brain structure. We tested this framework on the developing human connectome project (dHCP) dataset with 97 patients that were previously categorized by severity. Our framework demonstrated the model’s ability to identify and distinguish subtle morphological signatures present in brain structures. Normal and abnormal neonatal brain scans can be distinguished with reasonable accuracy, correctly categorizing them in up to 83% of cases. Most critically, new brain anomalies originally missed during the radiological reading were identified and corroborated by a neuroradiologist. This framework and our modeling approach demonstrate an ability to improve prognostication of neonatal brain conditions and are able to localize new anomalies.

## Introduction

There is a myriad of conditions that can impact the brain of a neonate which are critical to address at an early stage to improve the neonate’s quality of life and avoid significant health risk. One such example is neonatal encephalopathy (NE), a clinically defined syndrome leading to physiological and neuorological abnormalities, and is associated with significant lifelong consequences^1^. NE can often result in cerebral palsy (CP), which can be reliably diagnosed only at 18–24 months of life. CP has one of the highest disease burdens, and it is estimated that the lifetime cost for all U.S. patients with CP is $15 billion^2,3^. The occurrence of brain injuries that result in neurological disorders like NE and CP are believed to primarily happen before birth, with some cases occurring after birth. Therefore, the early identification of brain injury through novel biomarkers may lead to improved neurobehavioral outcomes^4^.

Neonatal brain MRI scans are most often utilized for diagnosis of NE, to determine the extent of abnormality. Recent technical improvements in high-field MRI have made abnormalities more detectable to the radiologist, however, radiologists are prone to perception errors at a rate of 3–6% when findings are not identified. They are also prone to interpretation bias and differ in their opinion of what constitutes critical brain injury^5–9^. There are potential limitations associated with the human eye’s ability to observe the combinatorial patterns of injury in different areas of the brain, particularly in a three-dimensional context. To address those limitations, several machine learning and deep learning approaches have been proposed to assist neuroradiologists in reducing observer dependence, address under-utilization of numerical data, and improve diagnostic accuracy^10,11^. However, there is still a gap in literature focused on state-of-the-art prognostication methods for neonatal MRI brain scans, and there are inherent challenges associated with analyzing neonatal brains, namely, the rate of brain tissue development in neonates (brain volume, myelination), as well as limitations associated with image acquisition (motion artifacts, for example)^12^.

Deep learning models have shown great promise in medical imaging applications, demonstrating significant improvements over previous modeling efforts, though they have primarily taken *supervised* learning approaches^11,13^. One common application of deep learning in medical imaging is that of brain tissue segmentation, where a variety of modeling approaches have found success. The Brain Tumor Segmentation (BraTS) competition has consistently led to cutting edge developments in deep learning architectures conducive to the analysis of medical image data^14–17^. Autoencoder (AE) networks have recently been used for anomaly detection in medical images, including the detection of chronic brain infarcts, though current approaches tend to incorporate the AE architecture to assist the supervised learning process of other parts of the network^10^. Both AE’s and Variational Autoencoders (VAE) have shown promise in their ability to learn generalized latent features and ResNet-based VAE’s, specifically, are commonly used for medical imaging analysis^18–21^. Supervised approaches typically require high quality labeled data, which are often unavailable in clinical settings, leading to the need for unsupervised approaches. A successful example of unsupervised modeling in the medical imaging space applied a VAE to PET data of Alzheimer’s disease patients and was able to effectively differentiate between cognitively normal and Alzheimer’s patients by recognizing a higher reconstruction error in anomalous regions of the scans^22^.

All aforementioned analyses utilized adult brain scans. Several works have been applied to the neonatal brain, both with deep-learning and non-deep-learning methods. Multivariate analysis (MVA) has shown promise in its ability to distinguish between tissues, anomalies, and structural patterns in neonatal brain scans that may indicate the presence of an underlying condition^23^. One classification approach to neonatal brain imaging involves the use of machine learning to extract features from the images, which are then refined using feature selection, after which classification is applied using the refined features^24^. This approach was able to effectively diagnose acute bilirubin encephalopathy in a supervised learning context.

In this work, we hypothesize that an unsupervised, model-based approach to analyzing neonatal MRI brain scans will detect new anomalies missed by neuroradiologists. Herein we demonstrate a set of essential image preprocessing steps for model consumption, as well as deep learning based autoencoders for identifying anomalies in neonatal MRI brain scans to help highlight significant biomarkers and reduce interpretation biases on the part of neuroradiologists. We present an approach to reconcile model outputs with real world interpretation and generate biomarkers that can prognosticate death and disability, including CP.

## Methods

In this study, we compared AE and VAE-type architectures to identify an effective model for detecting anomalies within the neonatal brain. We utilized image preprocessing methods to ensure homogeneity in the dataset, and postprocessing to ensure the outputs of our models can be interpreted by a radiologist. Our methods were as follows:

### Anomaly detection using autoencoders

**Figure 1:**
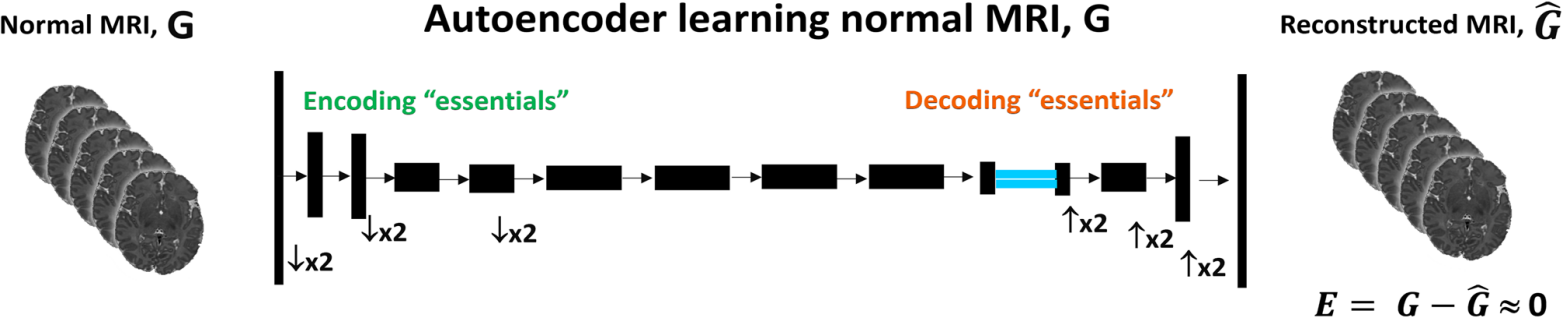
Autoencoder (AE) network employs an encoder-decoder structure for learning. Encoders sequentially abstract or compress (down-sample, $\downarrow \times 2$) the input data, *G* to find the most effective feature maps while decoders sequentially amplify or expand (up-sample $\uparrow \times 2$) the resulting feature maps to reconstruct output data, ${\hat{G}}$ that minimizes the error between *G* and ${\hat{G}}$, $E = G - {\hat{G}}$. Each block within the AE consists of multiple convolutional neural network layers, rectifiers, and normalization layers that are tuned during learning using MRI data from seemingly normal populations.

We chose to use 3D AE architectures as our baseline for this manuscript. We trained these models using MRI data from seemingly “normal” cases, to learn the common critical geometric and structural characteristics of a brain, minimizing recreation error of the regenerated brain volume (see Fig. 1). As such, when presented with the MRI of a CP, death, or otherwise abnormal patient, the regions with high AE reproduction error form the basis for the development of biomarkers for early identification of at-risk newborns for motor deficits. We used a 3D approach in order to preserve cross-slice voxel information.

A core component of the proposed model was the ResNet block^25^. This block is built of two consecutive sets of normalization, activation, and 3D convolution layers, which are then added to the original input (bypass) at the end. This allows the model to learn features of the input through convolution while preserving additional global information. These ResNet blocks have shown promise in the analysis of medical imaging data^15^. For our standard 3D AE (denoted as *std_level of compression*), we tested model architectures that compressed the original image to 8x8x8 (*std_8x8* ) and 16x16x16 (*std_16x16* ) volumes.

### Anomaly detection using variational autoencoders

**Figure 2:**
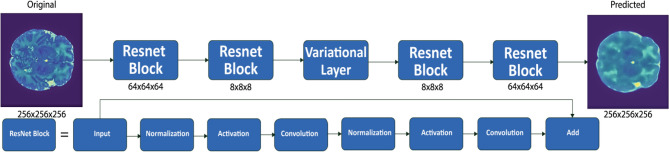
Our modeling architecture used a sequence of ResNet blocks. The standard AE implementations do not include the inner variational layer, while the VAE implementations do. It is in this variational layer where we vary our sampling parameters to determine which number of samples optimizes for detecting anomalies.

The base architecture and ResNet blocks for the VAE were the same as defined for the AE models. In the VAE case, we also added a variational layer (denoted as *vae_level of compression*) to sample from a constrained multivariate latent distribution. While exploring the same levels of compression for the 3D AE, we also varied the number of nodes in the latent space, examining node counts of 256 (*vae_8x8_256* , *vae_16x16_256* ), 512 (*vae_8x8_512* , *vae_16x16_512* ), and 1024 (*vae_8x8_1024* , *vae_16x16_1024* ). The base architecture of our VAE approaches are otherwise the same as the AE approach. A generalized illustration of our proposed model architecture can be found in Fig. 2.

### Data description

A collection of T2 weighted MRI brain scans from the developing Human Connectome Project (dHCP) were used to train and test the proposed models^26^. Every scan in this dataset had been reviewed by dHCP radiologists, who agreed on a score ranging from 1 to 5, where 1 represents a normal appearance for the patient’s age and 5 represents incidental findings with possible/likely significance for both clinical and image analysis (e.g. major lesions in white matter). Given the non-specificity of the ordinal classification in scores 2–4, we chose to analyze only the scans with scores 1 or 5, with score 1 being described as “normal” and score 5 described as “abnormal” henceforth. In total, 80 normal brain scans were used to train the model, 9 normal brains were held out as for validation, and 8 abnormal brain scans were used to test the model’s ability to learn structures that define a normal brain and identify anomalies in the neonatal brain. The patients in this dataset have a gestational age ranging from 24 to 45 weeks, though the vast majority of patients were term births. All samples have been processed in accordance with the dHCP structural pipeline to ensure uniformity^27^.

### Data preparation

Our preprocessing pipeline was applied on top of the images that were already preprocessed by dHCP’s structural pipeline. To further ensure a relatively homogeneous population of images, we applied preprocessing steps provided by the Advanced Normalization Tools (ANTs) to prepare our data for modeling^28^.

We began by applying skull stripping to remove the skull and any extraneous tissue from around the brain^29^. Next, we applied bias field correction to reduce any lack of uniformity in the same tissues (e.g. white matter) across the sample^30^. Finally, we resized each image in our sample set to 256 $\times$ 256 $\times$ 256 pixels (image dimensions were inconsistent from scan to scan) and applied a fast affine image registration step to ensure the brain volume appeared within the same location in each image^31^. Each image was registered against a representative normal sample from the dHCP dataset - all images, whether normal or abnormal, were registered against this same reference brain.

All models were trained using a desktop with an AMD Threadripper 3960$\times$ with 24 cores, 128GB of RAM, and an NVidia Titan RTX GPU. Training each model took approximately 2 hours to complete, over a total of 50 epochs. The code for our approach is available on Github and the associated data can be requested through dHCP’s database.

### Analysis framework

We used two metrics to quantify the effectiveness of our models in their ability to distinguish between normal and abnormal brain scans. We used mean squared error (MSE), where $MSE = \frac{1}{n} \sum ^{n}_{i=1} (Y_i - {\hat{Y}}_i)^2$, to quantify the difference between the original image and the recreated image. The second metric we used was a measure of the clustering of errors in the output image, using slice-wise 2D connected components^32^. Collectively, these metrics are intended to ensure that anomalous pixels are well clustered, and that normal brain structure is being distinguished from abnormal brain structure. To add additional interpretability to the anomalous pixels and clusters, we also generated banded bins of standard deviations across all architectures and sample sets. For pixel counts, we calculated the Z-score for the residual of each pixel in a brain volume, and measured the percentage of pixels that fall within each of the following ranges of values: −infinity to −4, −4 to −3, −3 to −2, 2 to 3, 3 to 4, and 4 to infinity. Similarly, we measured the average size of clusters that fall into each of the aforementioned standard deviation ranges. These metrics help define the magnitude of reconstruction error in both the positive and negative direction, acting as a proxy for the contrast of the tissue being reconstructed. For example, for T2 scans, positive standard deviations would indicate reconstruction error in high contrast regions like fluid space, while negative standard deviation suggest reconstruction error in low contrast regions like white matter. We also tested the abilities of our architectures to distinguish between train and test sample sets by using Receiver Operating Characteristic (ROC) curves. We used sample-wise total MSE to generate our decision boundaries, and radiology scores 1 and 5 (normal and abnormal) as our target class.

Further validation was conducted to assess each model’s ability to localize anomalies found within the brain. We generated a normalized heat map of errors comparing original and recreated images. These heat maps showed the size of the error as a Z-score at each voxel, and helped visually identify regions that were proposed as anomalous. A sample of normal images and all abnormal cases were reviewed by our neuroradiologist (S.M.) to determine the clinical validity of highly anomalous regions.

### Ethics declarations

This study does not involve human subjects.

## Results

### Autoencoder and variational autoencoder model results

**Figure 3:**
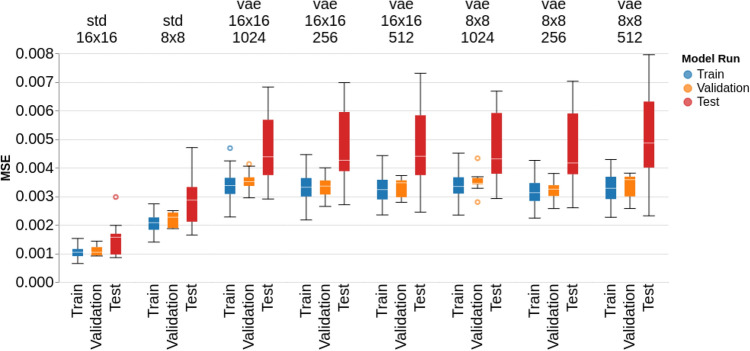
Train (normal), Validation (normal), and Test (abnormal) MSE’s across each model architecture.

The AE models demonstrated an ability to recreate brains from a compressed feature representation, with *std_16x16* and *std_8x8* showing an average training MSE of 0.001 and 0.002, respectively, in Fig. 3. Test MSE’s for these models were 0.001 and 0.002 respectively. All VAE models showed higher train errors, ranging between 0.003 and 0.004, as compared to the AE models. Similarly, test MSE’s for the VAE models were also higher, ranging between 0.004 and 0.005. Across all VAE models, we see a wider interquartile range of MSE values across the test dataset, as compared to the AE models. We also see greater divergence between the train and test sets for our VAE architectures, with the median test MSE’s being significantly greater than the median train MSE’s. Across all AE and VAE architectures, there is no significant difference between MSE’s for the normal train and validation samples.

**Figure 4:**
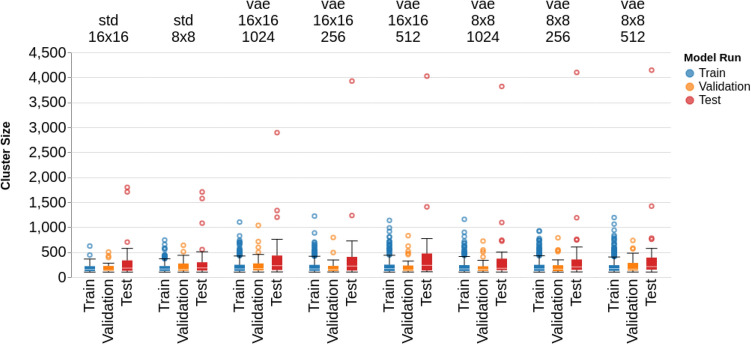
Train (normal), Validation (normal), and Test (abnormal) cluster sizes (in pixels) across each model architecture.

In Fig. 4, the cluster sizes of anomalous pixels across all tested architectures are shown. The AE architectures show average train cluster sizes of 11 and 16 for the *std_16x16* and *std_8x8* architectures, while the average test cluster sizes were 17 and 23, respectively. For the VAE architectures, the average cluster size ranged from 20 to 25 for the training samples, and 32 to 38 for the test samples. The range of values across cluster sizes is smaller than for MSE’s, though the VAE architectures consistently show a wider range of test cluster sizes than the AE architectures.

In Supplementary Figures S1 and S2, we see the banded percentage of pixel residuals that fall into each range of standard deviations across all modeling architectures. The AE architectures have the largest number of pixels by volume in the 2 to 3 standard deviation range, while the distribution of pixels in the positive direction across all other ranges and architectures are more consistent from model to model. There does not appear to be as large of a variation in pixel percentages across the negative standard deviation ranges. Similarly, for our banded cluster sizes in Supplementary Figures S3 and S4, we see many clustered pixels in the 4 to infinity range of standard deviations, particularly for the VAE architectures. We also see a greater separation between train and test clusters in that band for the VAE architectures. In the negative direction, we see larger cluster sizes in the -4 to -infinity range for all VAE architectures, although the difference is less pronounced than the clusters in the 4 to infinity range. We generally see smaller clusters overall for the negative standard deviations.

Based on the ROC curves in Supplementary Figures S5 and S6 we note that the 8x8 architectures have higher areas under the ROC curve (AUC) as compared to their 16x16 counterparts. The highest AUC reported is for *vae_8x8_1024* at 0.83, with the lowest being for *std_16x16* at 0.74.

### Clinical and visual results

Given the unsupervised nature of our modeling approach, it is important to consider the clinical and visual aspects of the recreated brain scans in order to understand their abilities to act as a radiologist’s aide. We used the aforementioned residual map overlays to help identify anomalies within the brain.

**Figure 5:**
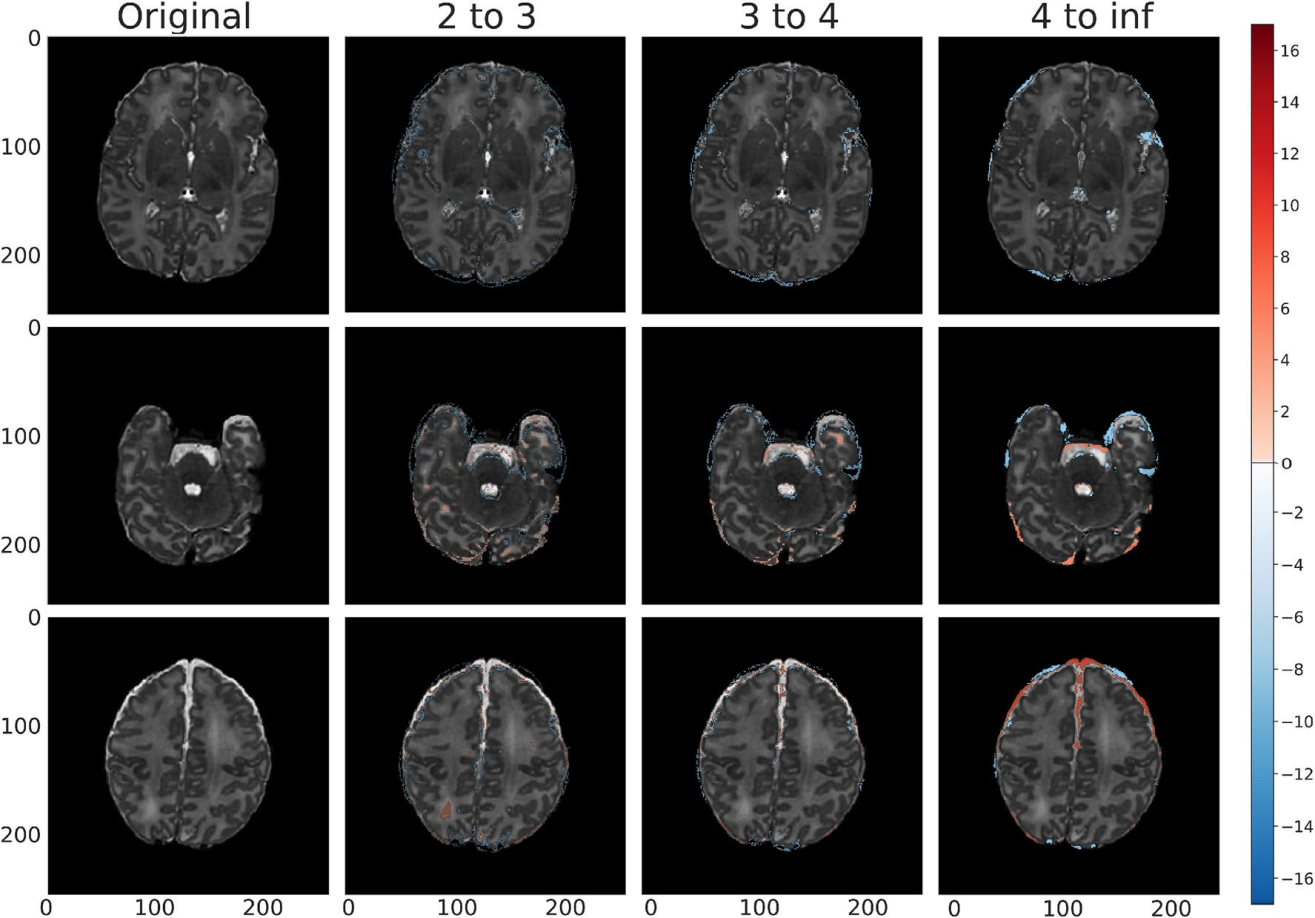
Train (labeled normal, gestational birth age of 39 weeks) anomaly detection map for *vae_8x8_512* across each standard deviation range. The first column shows slices without anomaly overlays, and the subsequent columns represent the model outputs overlayed by range of absolute standard deviations for clarity. We consider standard deviations that are within the following ranges: 2 to 3, 3 to 4, and 4 to infinity, where the higher standard deviations indicate a greater potential severity of anomaly (divergence from normal appearance). Positive standard deviations of the reconstruction error map are indicated in shades of red and negative standard deviations are indicated in shades of blue.

**Figure 6:**
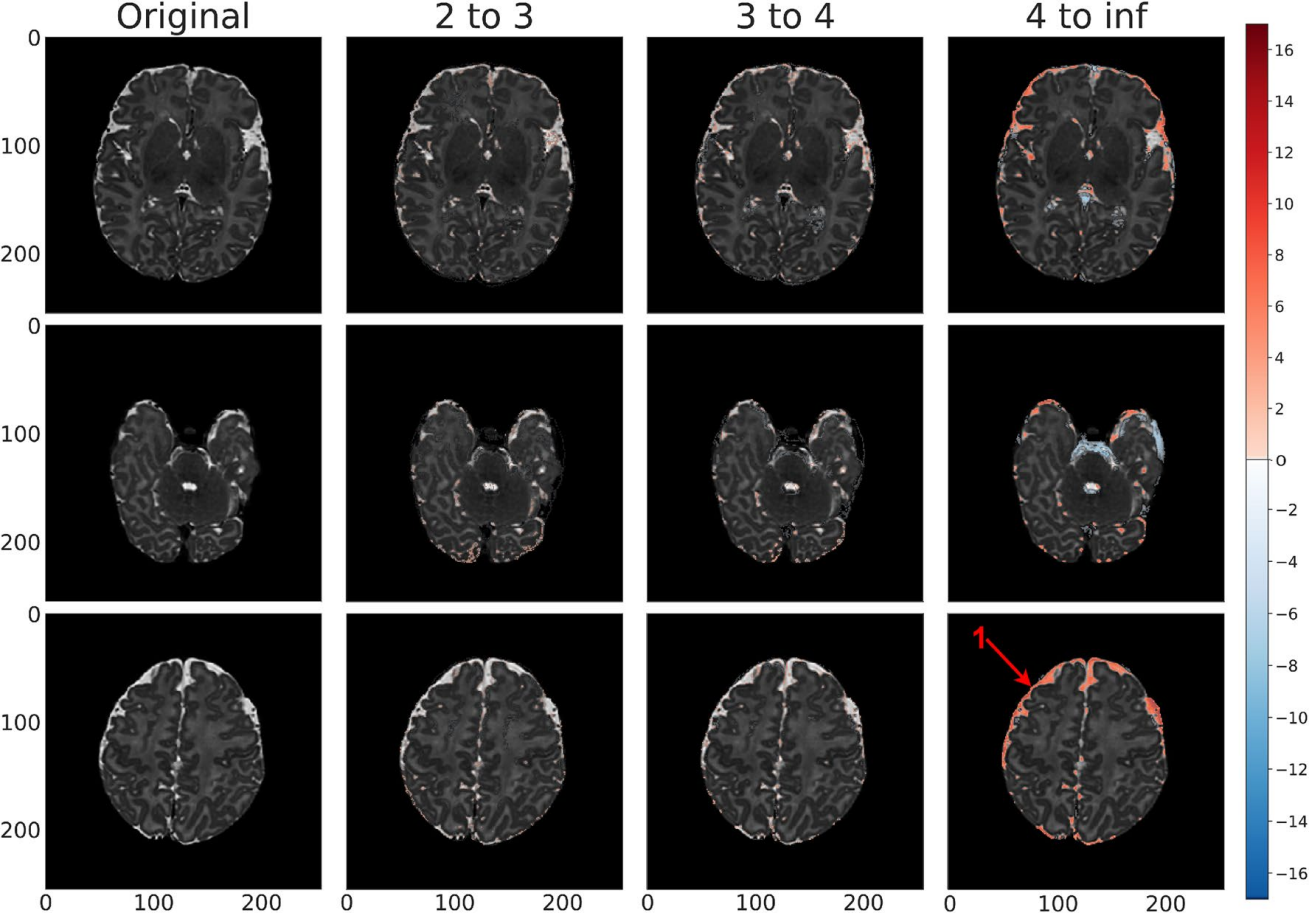
Validation (labeled normal, gestational birth age of 40 weeks) anomaly detection map for *vae_8x8_512* across each standard deviation range. Positive standard deviations of the reconstruction error map are indicated in shades of red and negative standard deviations are indicated in shades of blue. The peripheral highlighted regions represent variation in normal fluid spaces in and around the brain parenchyma (arrow 1).

**Figure 7:**
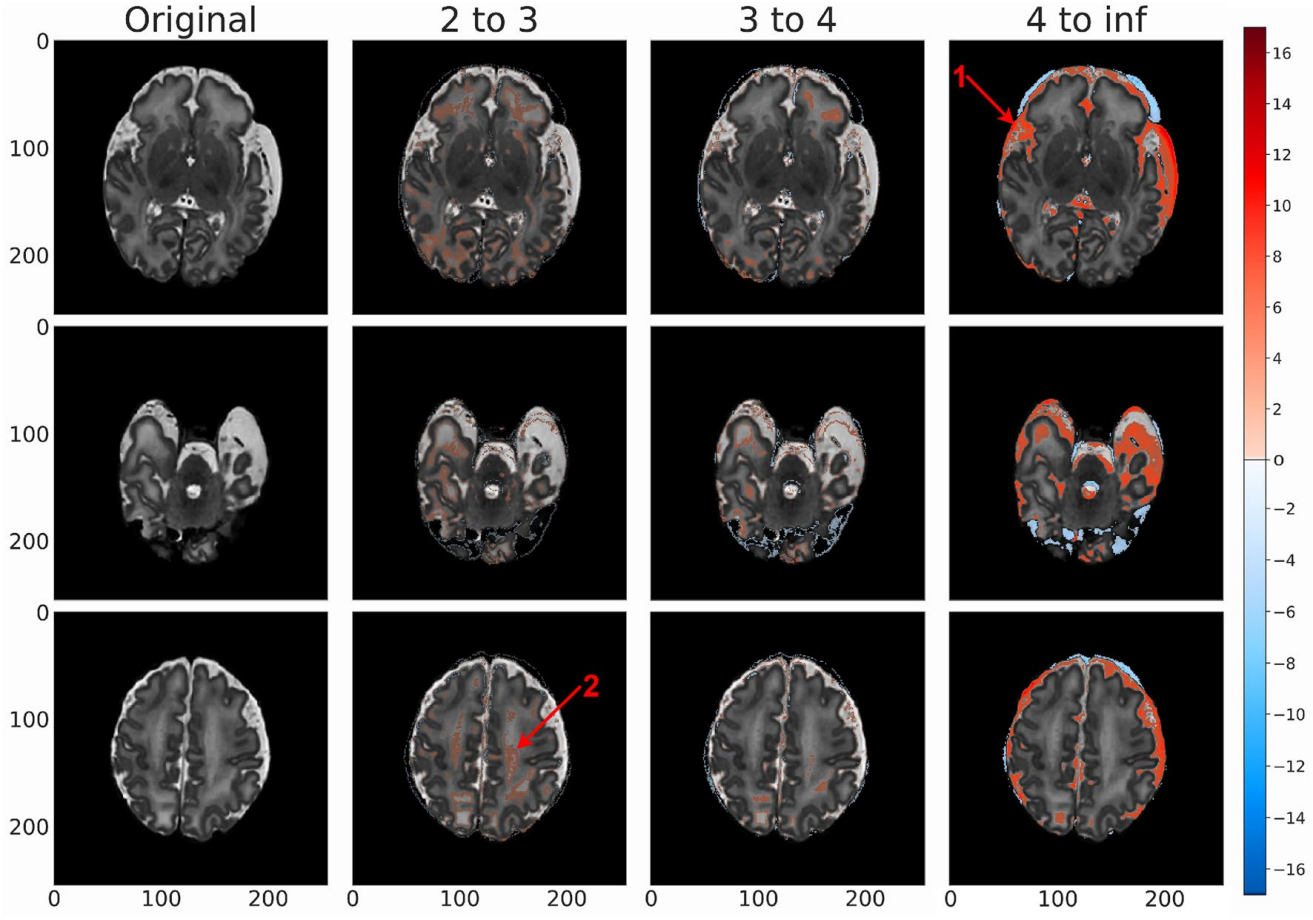
Test (labeled abnormal, gestational birth age of 37 weeks) anomaly detection map for *vae_8x8_512* across each standard deviation range. Positive standard deviations of the reconstruction error map are indicated in shades of red and negative standard deviations are indicated in shades of blue. In this example, the peripheral and deep white space as well as asymmetric fluid space ($3^{+}$ standard deviations, arrow 1) is indicative of volume loss in the left hemisphere. We also see hemorrhage along the tentorium and occipital region (2–4 standard deviations, arrow 2).

**Figure 8:**
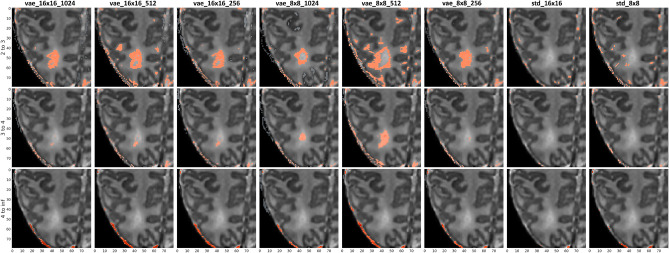
This specific anomalous region was identified by our radiologist in an MRI scan of a term birth (39 weeks) subject that was labeled as normal by dHCP. Our model identified an anomaly in the right occipital lobe, which was confirmed by our radiologist. Here we isolate the region across each of our model architectures for the same standard deviation ranges used in Figs. 5, 6 and 7 to determine which approach was most resilient to an anomaly in what was otherwise considered a normal brain.

In Fig. 5 we saw the anomaly overlay for a training example reproduced by our best model (*vae_8x8_512*, see Discussion and Conclusion for details). We took a similar banded approach in this figure as with the banded pixel percentages and clusters to better distinguish the severity of the model’s detected anomalies. We also visualize the three slices from the same sample - the middle slice of the volume, and slices 64 units in each direction from the middle slice. The highlighted region within the Sylvian fissure area mostly visible in standard deviation levels $4^{+}$, appeared to be captured mainly due to the existence of asymmetrically prominent fluid space. This is likely due to a structural anomaly rather than a slightly tilted head due to its existence in consecutive slices. Although this brain was initially classified as healthy, it appeared to have a prominent class 1A injury (i.e. minimal cerebral lesions without basil ganglia or thalamic involvement or internal capsule) using the previously published classification scheme for neonatal encephalopathy^33^.

Similarly, Fig. 6 showed the anomaly detection maps generated by the proposed framework on a brain scan within the validation set. Our framework classified this brain as normal and there are minimal highlighted regions or noise in the anomaly map. This is consistent with the radiologist labeling of this sample, as provided by dHCP.

Figure 7 showed the anomaly detection maps generated by our proposed framework on an abnormal brain. Compared with seemingly normal T2 scans as shown in Figs. 5 and 6, the abnormal scan had higher positive and negative errors (i.e. proposed anomalies) in ventricles and white/gray matter regions, indicating biophysical deviation from a normal signature. This particular scan consists of significant cortical asymmetry along with excessive fluid space. The proposed framework was able to detect the anomalous temporal occipital white matter area in consecutive slices at standard deviation levels 2–3 and 3–4. The highlighted region within the frontal lobe at standard deviation levels 2–3 is consistent with sub-cortical lamination. In addition, the excessive fluid and asymmetry within the Sylvian fissure area was identified at standard deviation level $4^{+}$. The proposed framework was further able to identify the hemorrhage along the tentorium and occipital lobe parenchyma which was mostly visible in standard deviation levels 3–4 and $4^{+}$. The proposed framework also identified anomalies in the fluid space surrounding the brain - the fluid assymetry indicates parenchymal volume loss. As a result, this scan can be classified as a class 1B injury (i.e. more extensive cerebral lesions with no involvement of basal ganglia or thalamus, anterior limb of the internal capsule, posterior limb of the internal capsule, or watershed infarction).

The same region of a subject is highlighted across all modeling architectures in Fig. 8. Based on dHCP’s radiologist score, this brain was identified as normal, and as such, was used in the training process. Despite the dHCP label, our proposed framework flagged this specific region as abnormal, mostly visible at standard deviation levels 2–3 on several different model architectures. The same highlighted region could also been seen in the peripheral white matter area at the same standard deviation levels, and appears asymmetrically brighter in consecutive scans.

## Discussion and conclusion

We demonstrate for the first time the feasibility of an unsupervised approach to identifying anomalies in neonatal MRI brain scans. We trained and validated autoencoder models that demonstrated an ability to recreate normal brain structure and identify anomalous regions in abnormal brains. We also demonstrated clinical interpretation of the model outputs and found promise in the framework’s ability to act as a support tool for radiologists and identify anomalies that were not previously detected.

In typical approaches to modeling, similarity between train and test loss values can indicate that a model has generalized well to unseen data. In our case, we seek a greater divergence between normal and abnormal samples, indicating that anomalies are being identified in abnormal brains. Similarly, it was critical that train and validation metrics did not diverge significantly to ensure the approaches generalized well to normal brains that were not included in the training process. The AE models demonstrated an ability to effectively recreate brains from a compressed feature representation. However, when considering the AE results on the test (abnormal) samples, both models did not have a significant difference in metrics between train and test samples. As such, the AE models were not conducive to effectively identifying abnormalities in brain scans. These findings suggest that our AE architectures are learning to compress and decompress MRI brain scans without learning to replicate the input brain scan as closely as possible.

The VAE models were able to more effectively distinguish between normal and abnormal brains and demonstrate an ability to localize anomalous regions in an unsupervised fashion, with *vae_8x8_512* showing the largest difference between train and test metrics. The results in Supplementary Figures S1, S2, S3, and S4 highlight some important benefits of the VAE architectures. A higher number of pixels fell into the “4 or more” abnormality range (i.e. standard deviation level), suggesting these models are finding highly anomalous regions within the test set. We also see a significant increase in the average cluster size in our test data, indicating that the anomalous regions were well clustered and not isolated pixel noise. As previously noted, the 8x8 VAE architectures had the highest AUC as presented in Supplementary Figure S5. Though there are noted inaccuracies in the radiology score provided by dHCP, these architectures demonstrate a clear ability to distinguish between normal and abnormal brain volumes when treated as a classification problem.

While the aforementioned metrics confirmed the relatively strong performance of 8x8 VAE architectures, the metrics alone were not sufficient to pick an optimal model. In the clinical interpretation of our results, we found that *vae_8x8_512* is able to most effectively identify anomalous regions within the brain, and so we choose this architecture as the best of the ones assessed. This conclusion is best conveyed by Fig. 8. Based on dHCP’s radiologist score, this brain was labeled as normal and was used in the training process. Despite the dHCP label, our VAE models flagged the isolated region as anomalous, particularly in the 2–3 standard deviation range. The same highlighted region can also been seen in the peripheral white matter area and appears asymmetrically brighter in consecutive scans. The highlighted region was smaller in the 3 to 4 standard deviation range but *vae_8x8_512* maintained a strong signal in this range, supporting our conclusion that this is the best of our assessed models. These findings show promise in our approach and suggest that (1) our framework had the ability to aid radiologists for more accurate diagnosis, and (2) our modeling approach provides the ability to generalize normal structures within the brain despite the presence of abnormalities in the training dataset.

This is the first study in the newborn period to assess an unsupervised anomaly detection model system using VAE and deep learning on neonatal MRIs. We show that by using such a machine learning algorithm within our framework, normal and abnormal neonatal brain scans could be distinguished with reasonable accuracy, with up to 83% AUC in the ROC curves with a certain algorithm. More importantly, new brain anomalies originally missed during the radiological reading could be identified. Our AE models have the potential to be part of a decision support system for neuroradiologists evaluating neonatal brain MRI’s. It enhances radiological assessment by guiding the trained radiologists to certain overlooked areas of the brain which may result in optimizing diagnosis and treatment decisions. In addition, the identification of subtle morphological signatures through computational approaches that are beyond the capability of the human eye could serve as early biomarkers of key neurobehavioural events later in childhood. The AE method is advantageous due to the difficulties associated with collecting and labeling quality data.

Our framework works well with analysis of brain parenchyma as well as subarachnoid fluid spaces, generating both light and dark signals within these regions to highlight divergence from normalcy, as seen in Fig. 7. Even so, the following challenges were of note in our experimental process:

1. *Model interpretation of variability in fluid spaces* there is a significant amount of normal variability in the fluid spaces around the brains used in our sample dataset. While preprocessing and registration help address some of the issues associated with this variability, we found that a significant amount of the signal generated by our model focused on this fluid variability. Excluding these spaces from the training images could help “focus” the model more directly on the parenchyma.
2. *Noise* Preprocessing helped mitigate noise on the output, but there were still cases where individual pixels were being highlighted as anomalous without any adjacent anomalous pixels. We addressed this issue by using a standard deviation threshold to exclude low-error pixels, and validated the results of this thresholding visually and with our model metrics (MSE, clustering).

Our study also shows the need for improving anomaly localization. We surmise that some of the limitations with these models may be improved upon by allowing the model to consume and analyze multiple MRI modalities (T1, T2, Flair, etc), theoretically helping reduce the amount of attention on high contrast fluid spaces. Further experimentation and tuning of the preprocessing stages to address this issue could help improve the results further still. Incorporating a multi-task approach (e.g. autoencoding *and* classification) could help the model incorporate more global features from the dataset and reduce the attention placed on fluid spaces, though this would be sensitive to accurately labeled data.

Additionally, it is crucial to recognize that the dHCP data used to train the proposed framework in this study were obtained from individuals presumed to have normal brain development as well as an absence of encephalopathy. However, it is important to note that clinically it becomes impossible to confirm normal brain development or more importantly, that the newborn did not suffer from any hypoxic-ischemic insults. As a result, the presented network may have the ability to identify subtle or incidental abnormalities that differ from the trained structure, but these anomalies may not necessarily be related to NE. Consequently, additional testing is necessary to evaluate the effectiveness of the proposed network in detecting injuries associated with NE.

A possible drawback of the current approach is its limited generalizability to data from different institutions. We performed tests using non-dHCP data and discovered that the results were not suitable for inclusion in this manuscript. To address this issue, methods of harmonization across multiple institutions would typically be needed to ensure that MRI scans have a consistent visual appearance for modeling purposes.

Given our results, we notice the potential for future use in identifying conditions other than neonatal encephalopathy, including congenital anomalies, infections, and trauma. This architecture could also be used to recognize longitudinal normal brain development in order to recognize delays in myelination or abnormal myelination in leukodystrophies and metabolic disorders. Furthermore, the model could be be trained to recognize abnnormal sulcation patterns and areas of heterotropia and cortical dysplasia in patients with seizures.

Autoencoder-based models show promise for identifying anomalies in the neonatal brain. These models are able to effectively distinguish between normal and abnormal brains and localize anomalies. Our models also identified brain anomalies that were missed in the dHCP labeling process. We are now conducting future studies to develop neonatal MRI biomarkers from known outcomes of death and disability and hope more institutions would collaboratively join together in this effort. We speculate that algorithm based machine learning approaches for neonatal MRI scans have the potential to yield such biomarkers, which in turn, can help clinicians give a better prognosis to families and spur the development of new therapies for early intervention in targeted populations.

## Supplementary Information

Supplementary Information.

## Data Availability

The results published here are in whole or part based upon data generated by the developing Human Connectome Project (dHCP). The dHCP data is publicly available and can be downloaded at http://www.developingconnectome.org/. The source code for this manuscript is available at https://github.com/jraad/unsupervised_neonatal_anomaly_detection.
