# Supplementary material for: Unsupervised abnormality detection in neonatal MRI brain scans using deep learning
Source: Sci Rep. 2023 Jul 17;13:11489. doi: 10.1038/s41598-023-38430-0 (PMC10352269; doi:10.1038/s41598-023-38430-0)
Supplement: Supplementary file 1 — Supplementary Information. [file 41598_2023_38430_MOESM1_ESM.pdf]

Unsupervised Abnormality Detection in Neonatal MRI Brain Scans Using Deep Learning

Jad Dino Raad, Ratna Babu Chinnam, Suzan Arslanturk, Sidhartha Tan, Jeong-Won Jeong, Swati Mody

Supplementary Results

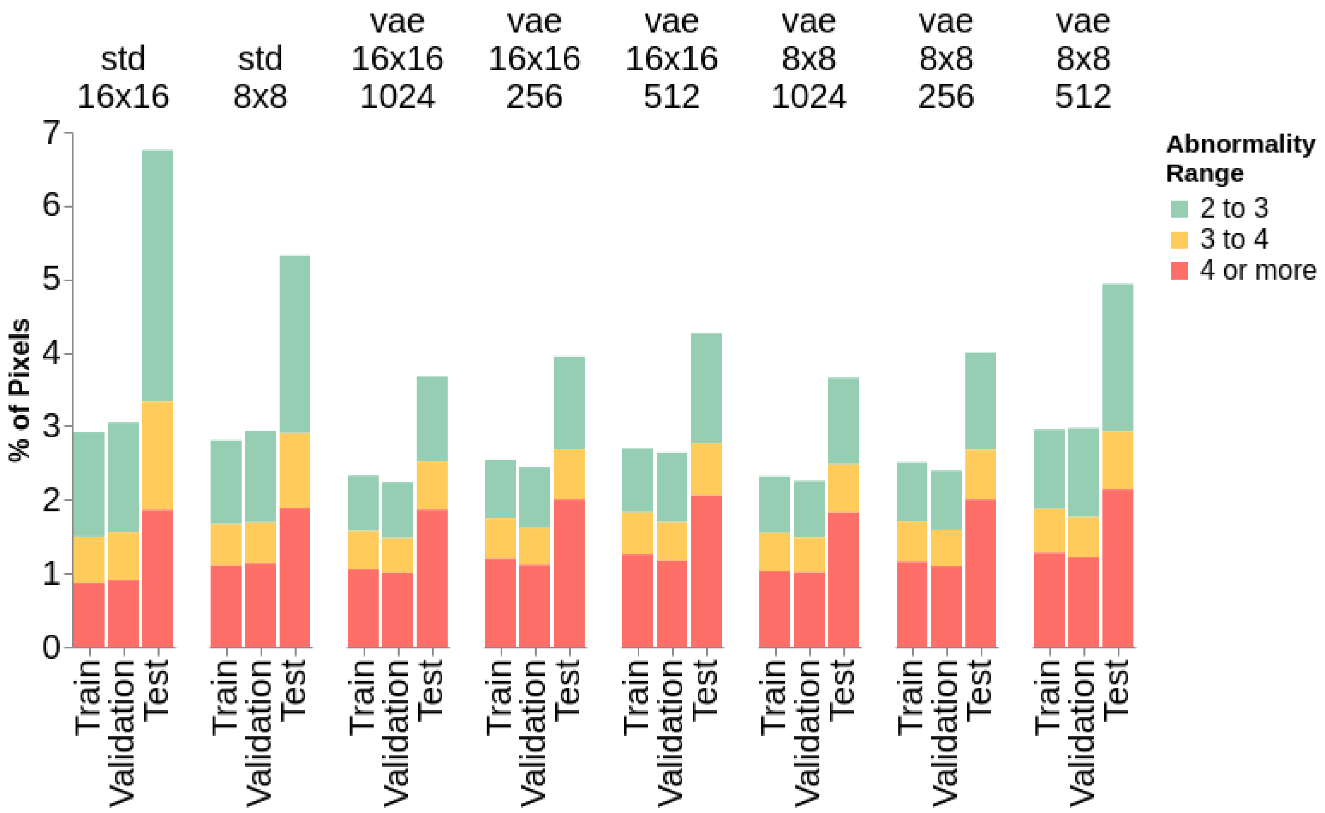

Figure S1. The percentage of pixels that fall within each range of positive standard deviations

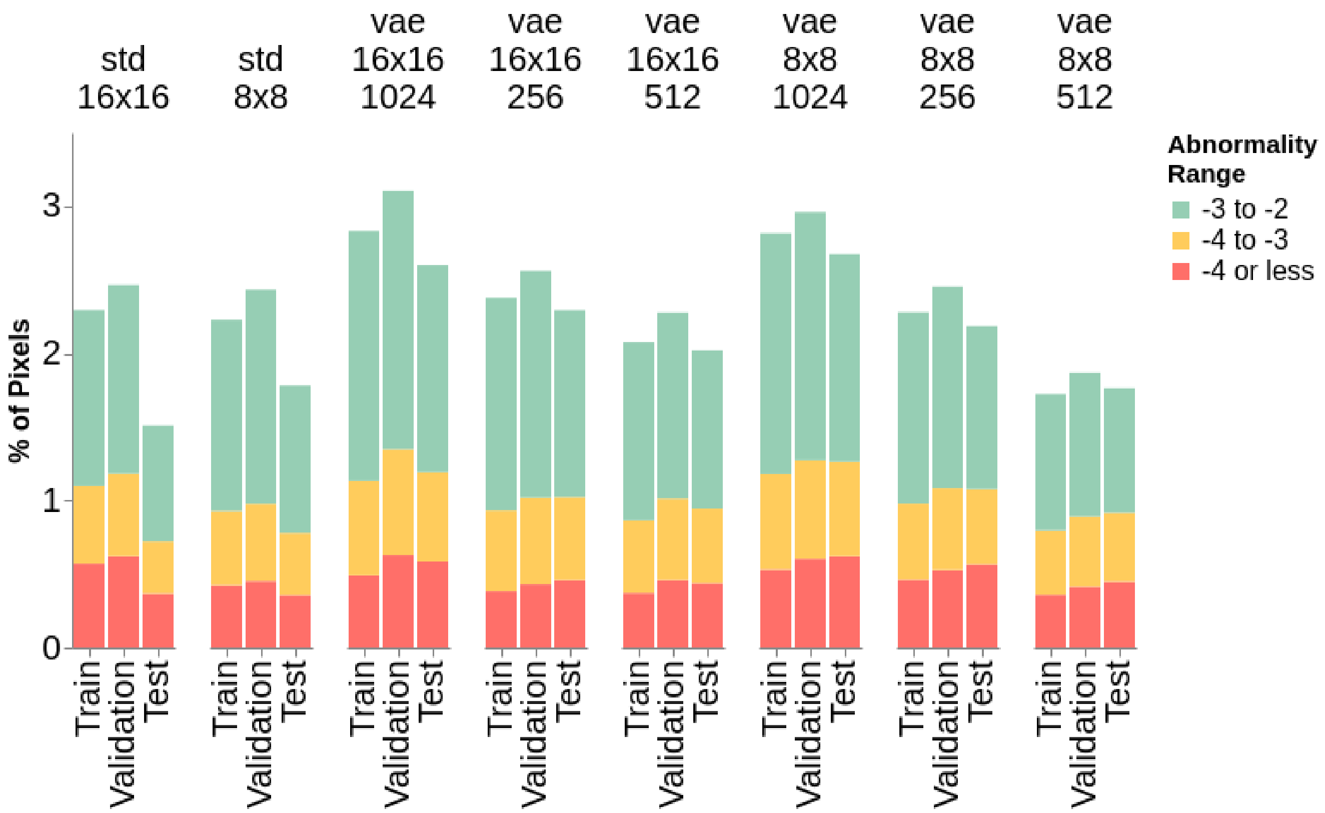

Figure S2. The percentage of pixels that fall within each range of negative standard deviations

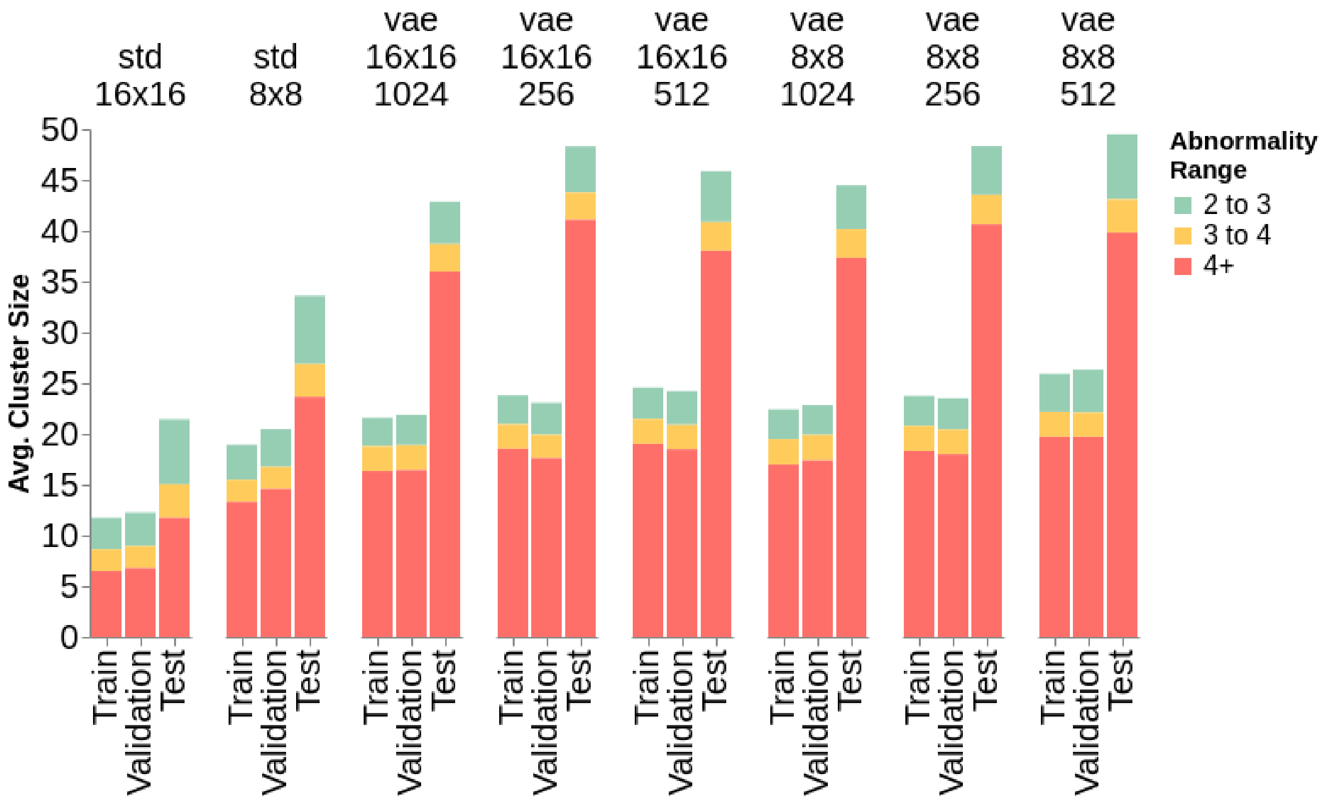

Figure S3. The average cluster sizes that fall within each range of positive standard deviations

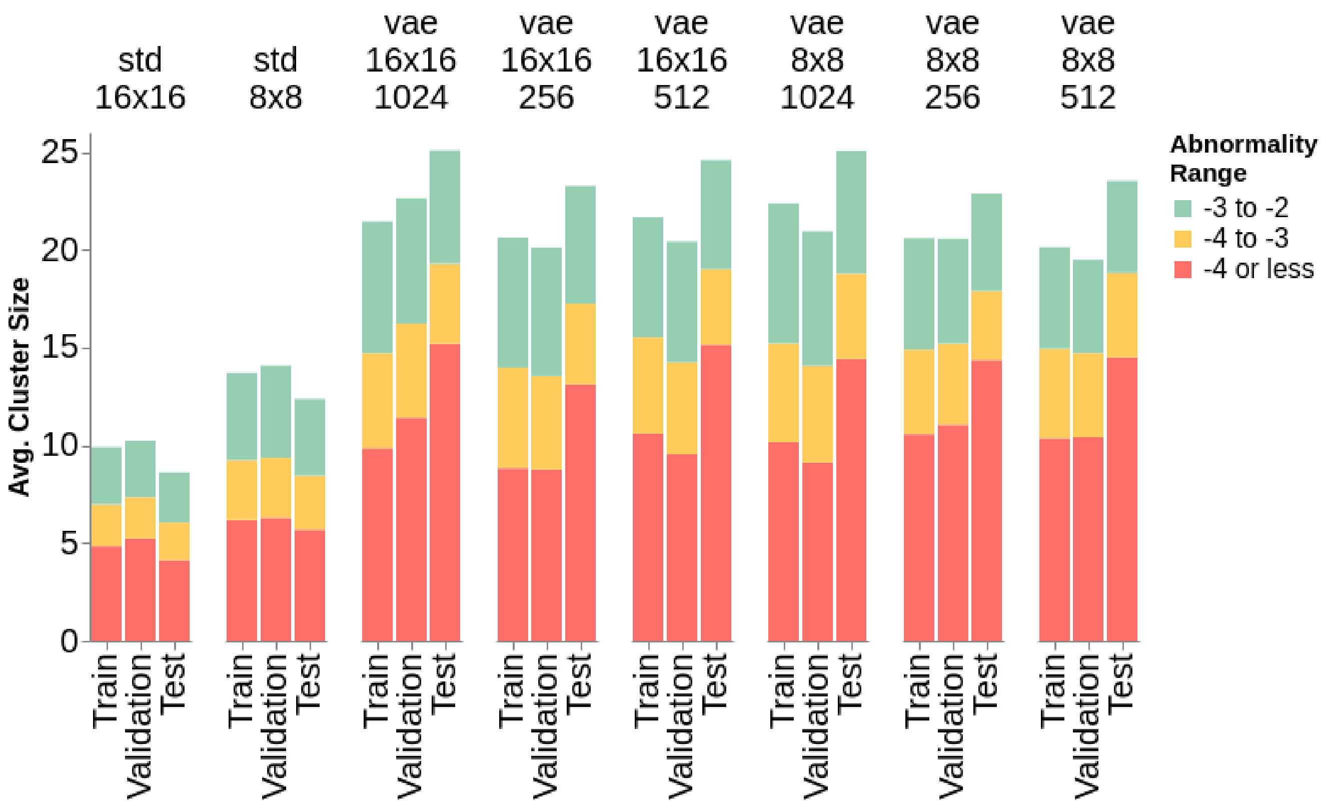

Figure S4. The average cluster sizes that fall within each range of negative standard deviations

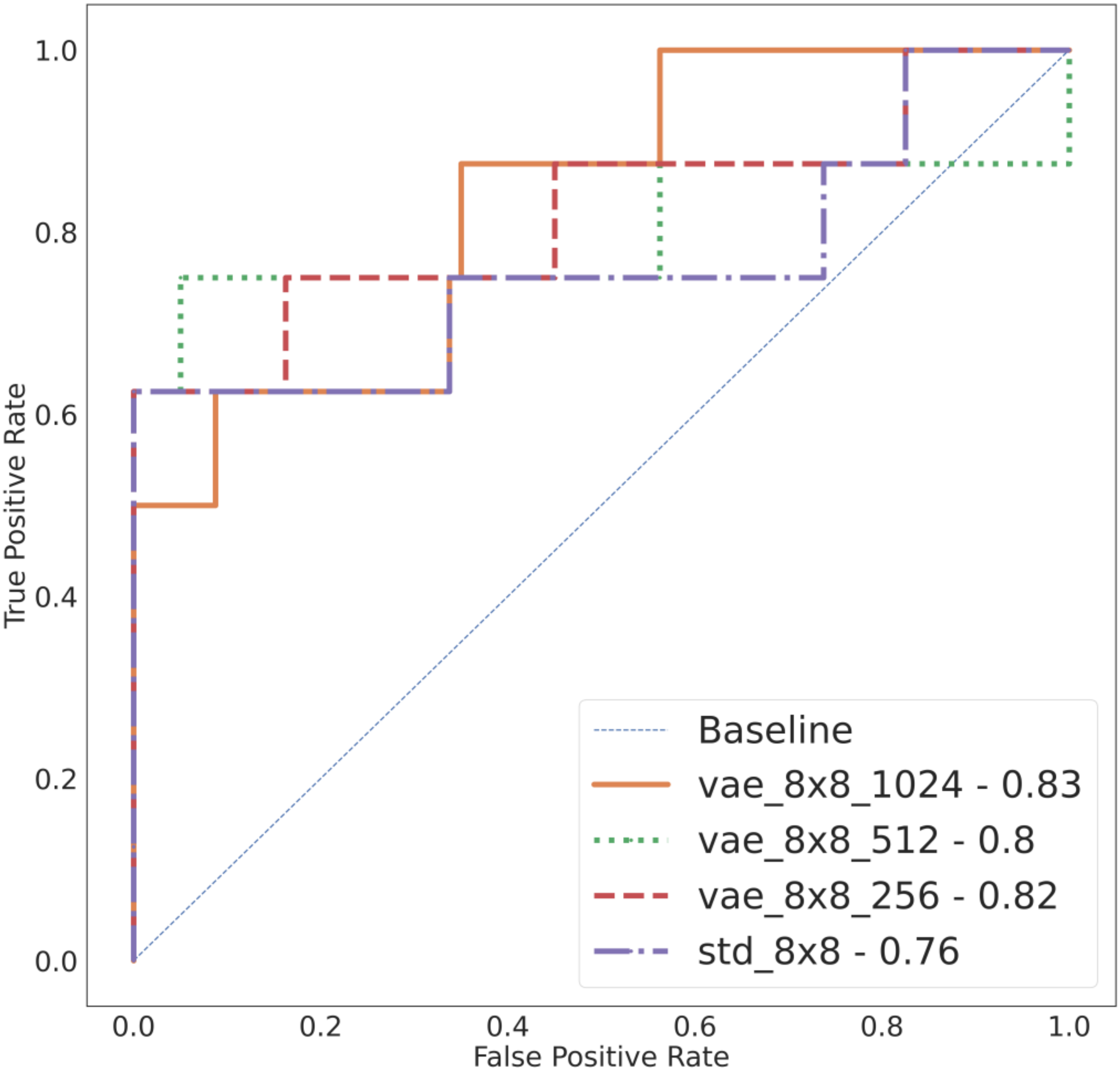

Figure S5. ROC curves for 8x8 model architectures (Area Under Curve (AUC) reported in legend)

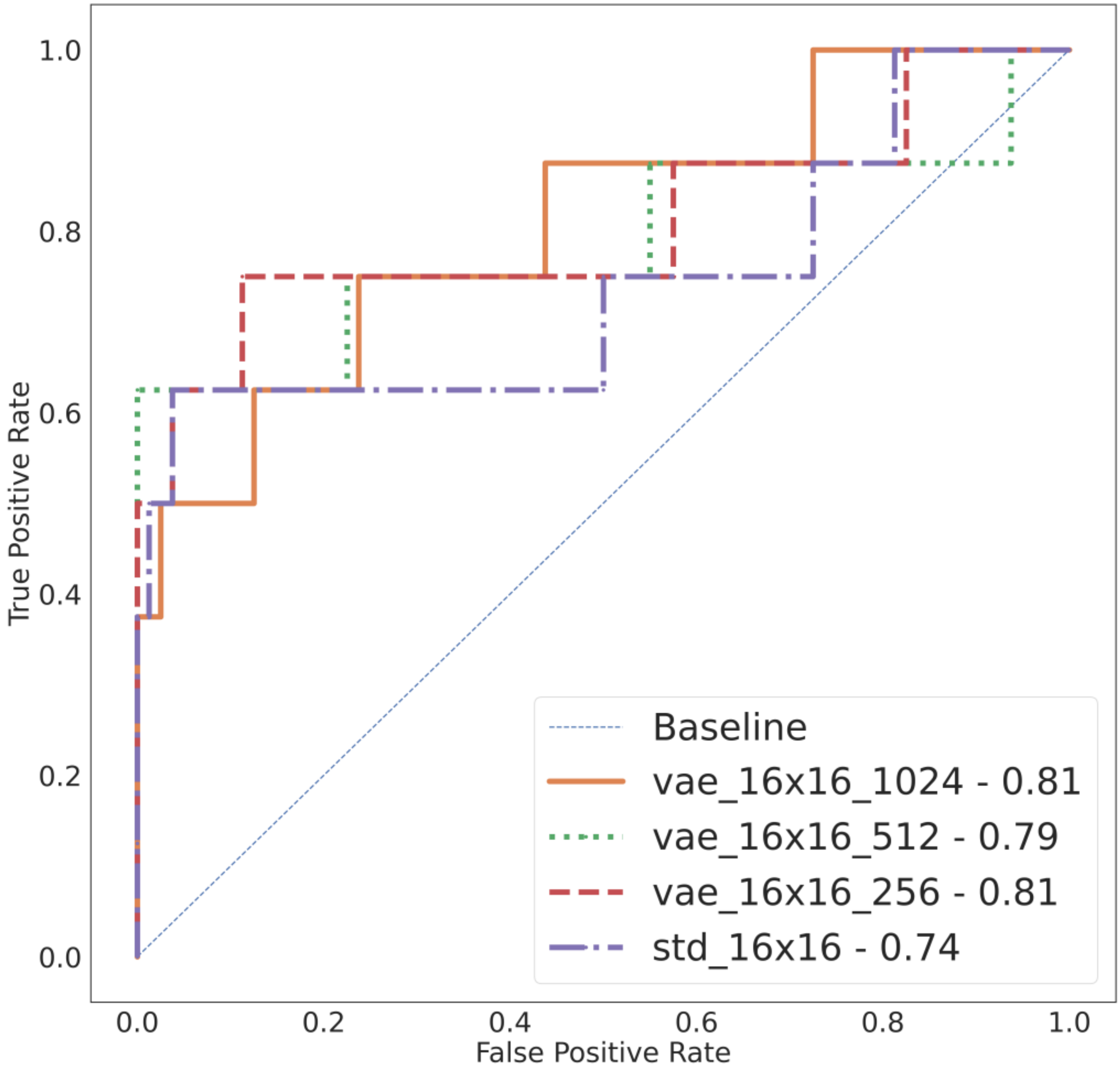

Figure S6. ROC curves for 16x16 model architectures (Area Under Curve (AUC) reported in legend)
